# Repurposing of Doramectin as a New Anti-Zika Virus Agent

**DOI:** 10.3390/v15051068

**Published:** 2023-04-27

**Authors:** Yujia Zhu, Minqi Liang, Jianchen Yu, Bingzhi Zhang, Ge Zhu, Yun Huang, Zhenjian He, Jie Yuan

**Affiliations:** 1 School of Public Health, Sun Yat-sen University, Guangzhou 510080, China; zhuyj28@mail2.sysu.edu.cn (Y.Z.);; 2 Key Laboratory of Tropical Disease Control (Sun Yat-sen University), Ministry of Education, Guangzhou 510080, China; 3 School of Chemistry, South China Normal University, Guangzhou 510006, China; 4 Zhongshan School of Medicine, Sun Yat-sen University, Guangzhou 510080, China; 5 School of Pharmacy, Guangdong Pharmaceutical University, Guangzhou 510006, China; 6 School of Basic Medical Sciences, Southern Medical University, Guangzhou 510515, China

**Keywords:** ZIKV, doramectin, antiviral agent, RdRp

## Abstract

Zika virus (ZIKV), belonging to the *Flavivirus* family and mainly transmitted by mosquitoes, causes a variety of adverse outcomes, including Guillain-Barré syndrome, microcephaly, and meningoencephalitis. However, there are no approved vaccines or drugs available for ZIKV. The discovery and research on drugs for ZIKV are still essential. In this study, we identified doramectin, an approved veterinary antiparasitic drug, as a novel anti-ZIKV agent (EC_50_ value from 0.85 μM to 3.00 μM) with low cytotoxicity (CC_50_ > 50 μM) in multiple cellular models. The expression of ZIKV proteins also decreased significantly under the treatment of doramectin. Further study showed that doramectin directly interacted with the key enzyme for ZIKV genome replication, RNA-dependent RNA polymerase (RdRp), with a stronger affinity (K_d_ = 16.9 μM), which may be related to the effect on ZIKV replication. These results suggested that doramectin might serve as a promising drug candidate for anti-ZIKV.

## 1. Introduction

Zika virus (ZIKV), discovered in Uganda in 1947 [1], is a positive-sense and single-stranded RNA virus belonging to the *Flavivirus* family. The transmission modes of ZIKV mainly include mosquito-borne transmission, mother-to-child transmission, sexual transmission, and blood transmission [2]. It is worth noting that ZIKV usually does not cause serious illness in adults [3], but during pregnancy, ZIKV is deleterious to the fetus, which is associated with fetal death, fetal growth restriction, and a spectrum of central nervous system abnormalities [4]. Thus far, ZIKV has spread to 89 countries and territories around the world [5]. In 2016, ZIKV infected nearly 0.4–1.3 million people in Brazil. In the same year, the World Health Organization (WHO) declared the ZIKV-related outbreak an international “public health emergency” [6]. Nonetheless, there are currently no vaccines or specific drugs available for ZIKV treatment.

ZIKV has more than 10,000 nucleotides which encode a single polymeric protein that can be cleaved into three structural proteins (Capsid, pre-Membrane and Envelope) and seven nonstructural proteins (NS1, NS2A, NS2B, NS3, NS4A, NS4B, and NS5) [7]. Notably, all viral proteins play an important role in the life cycle of ZIKV. Among them, NS1 induces ER perinuclear aggregation with an ultrastructure resembling the formation of the viral replication compartment [8]. Additionally, NS3 possesses RNA helicase, nucleoside, and RNA triphosphatase activities and involves both in viral RNA replication and virus particle formation [9]. Moreover, NS2B-NS3 functions as a hub for flavivirus replication complex assembly and regulates viral pathogenesis and host immune response [10,11]. Furthermore, NS5 is essential for the replication of the RNA genome of flaviviruses through mediating the synthesis of the viral genome, which is based on its methyltransferase (MTase) and RNA-dependent RNA polymerase (RdRp) activities [12]. Significantly, RdRp, a multi-domain viral enzyme, utilizes the associated metal ions on the RNA template to exert its polymerization effects, thereby promoting the synthesis of viral nucleic acids [13,14]. At present, many groups have conducted antiviral studies by targeting RdRp. For example, Takahashi et al. have developed a type of RdRp inhibitor that disturbs the interaction between RdRp and NS3 to repress virus replication [15]. In addition, it is worthwhile to develop antiviral drugs that interfere with the structural motifs or viral nucleic acid sequences of RdRp to inhibit its activity [16].

**Figure 1:**
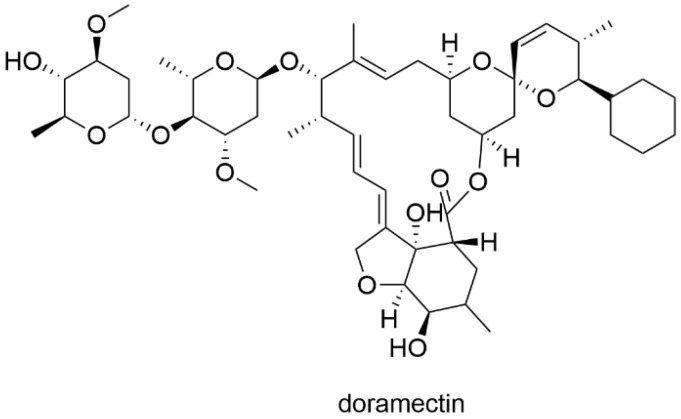
The structure of doramectin.

Doramectin, 25-cyclohexyl-5-O-demethyl-25-de(L-methylpropyl) avermectin A1a (Figure 1) [17], is a macrolide most widely used as a veterinary medication approved by the US Food and Drug Administration (FDA) for the treatment of animal parasitic infections [18]. Doramectin can also inhibit glioblastoma cell survival via the regulation of autophagy [19] and reverse the multidrug resistance (MDR) of breast cancer cells by inhibiting P-glycoprotein (P-gp) efflux [20]. In addition, ivermectin, a compound homologous to doramectin, has been previously reported to have great antiviral effects on a variety of RNA viruses, including SARS-CoV-2 [21], Venezuelan equine encephalitis virus (VEEV) [22], human immunodeficiency virus type 1 (HIV-1) [23], and multiple flaviviruses including dengue virus type 2 (DENV-2), Japanese encephalitis virus (JEV), and yellow fever virus (YFV) [24]. In this study, to identify effective anti-ZIKV agents, we conducted an in silico and functional screening of inhibitors against ZIKV RdRp using approved drugs, and identified doramectin with strong anti-ZIKV properties and low toxicity.

## 2. Materials and Methods

### 2.1. Cell Culture, Virus and Compounds

The human lung carcinoma cell line A549, African green monkey kidney epithelial cell line Vero, and human astrocytoma cell line SNB19 were obtained from the Cell Bank of the Chinese Academy of Sciences (CBCAS), Shanghai, China and cultured in DMEM (Invitrogen, Carlsbad, CA, USA) containing 10% fetal bovine serum (FBS) (GIBCO, Carlsbad, USA), 2 mM L-glutamine, 100 μg/mL streptomycin, and 100 units/mL penicillin (Invitrogen, Carlsbad, USA) at 37 °C under 5% CO_2_. All cell lines were authenticated by short tandem repeat (STR) fingerprinting at the Medicine Laboratory of Forensic Medicine Department of Sun Yat-Sen University (SYSU) (Guangzhou, China), and were found to be free of mycoplasma contamination. ZIKV ZG-01 strain (China, 2016, GenBank accession number KY379148), doramectin (catalog no.: HY-17035, batch no.: 109519) and ribavirin (catalog no.: HY-B0434, batch no.: 11970) were supplied in powder form by MedChemExpress LLC (Shanghai, China) with quality control documents.

### 2.2. Plaque Forming Assay

Plaque assays were performed to assess viral titers as previously described. Vero cells were seeded in a 12-well plate and cultured at 37 °C in 5% CO_2_ overnight. Then, the medium was removed and 5-fold serial diluents of virus were used to inoculate the cells. After 2 h, the supernatant was removed, and the cells were overlaid with a solution of 2.4% methyl cellulose in MEM supplemented and incubated for 7 days at 37 °C. Next, the cells were fixed with 4% paraformaldehyde for 30 min, washed, and stained with 2% crystal violet for 30 min. Finally, the number of plaques was counted, and the viral titers were reported as plaque-forming units per milliliter (PFU/mL). DMSO was used as a solvent control.

### 2.3. Cell Viability Assay

Cell viability was evaluated by the 3-(4,5-dimethyl-2-thiazolyl)-2,5-diphenyl-2H-tetrazolium bromide (MTT) assay. Briefly, cells were seeded in plates at a density of 6 × 10^3^ cells per well and incubated at 37 °C for 24 h, followed by exposure to the different concentrations of doramectin. DMSO was used as a solvent control. After 48 h, 5 mg/mL MTT solution was added to cells and incubated for an additional 4 h before removal. The insoluble formazan was solubilized with 160 mL of DMSO. The 96-well plates were read at 490 nm with an Enzyme standard instrument. The assay was performed in triplicate in three independent experiments.

### 2.4. Western Blotting Analysis

Protein expression was assessed using Western blotting as previously reported. The protein from tissues or cells were prepared by RIPA lysis buffer (Millipore, Bedford, MA, USA). Proteins were then separated by sodium dodecyl sulfate-polyacrylamide gel electrophoresis (SDS-PAGE), transferred onto a polyvinylidene difluoride (PVDF) membrane, and blocked with a solution of 5% non-fat milk in Tris-buffered saline buffer for 1 h. The membranes were probed with the following primary antibodies: anti-ZIKV E (GTX133314, 1:1000, GeneTex Inc., Irvine, CA, USA), anti-ZIKV NS5 (GTX133312, 1:1000, GeneTex Inc., Irvine, USA), and anti-GAPDH (60004-1-IG, 1:5000, Proteintech, Rosemont, IL, USA). Lastly, membranes were incubated with horseradish peroxidase-conjugated secondary antibody, and signals were visualized by enhanced chemiluminescence using a commercial kit (Thermo Fisher Scientific, Waltham, MA, USA) according to the manufacturer’s suggested protocols.

### 2.5. Immunofluorescence

Cells were fixed in 4% paraformaldehyde for 20 min and then permeabilized with 0.2% Triton X-100 (Sigma Aldrich, St. Louis, MO, USA) and blocked with 5% BSA in PBS for 1 h. Then, cells were incubated with an anti-ZIKV E antibody (1:500, BioFront Technologies, Tallahassee, FL, USA) for 12 h at 4 °C. A secondary antibody, Alexa 488-conjugated goat anti-mouse IgG (H + L) (A32723, Lifetechnologies, Carlsbad, CA, USA), was used for 1 h and 4′,6-diamidino-2-phenylindole nuclear stain (DAPI, 1:1000, Sangon Biotech, Shanghai, China) was used for counterstaining for 7 min at room temperature. The immunofluorescent images were taken with an inverted microscope (Carl Zeiss, Oberkochen, Germany).

### 2.6. Molecular Docking of ZIKV Proteins

The dockings of doramectin and ribavirin to ZIKV NS1 (PDB: 5K6K), ZIKV NS2B-NS3 (PDB: 7VXX), and ZIKV NS5 (PDB: 5TFR) were performed using the Molecular Operating Environment (MOE, 2010.10, Chemical Computing Group Inc., Montreal, QC, Canada) software with default parameters, and the docking site has been defined by the MOE Site Finder functionality. The results were generated with the method of Triangle Matcher (Placement) and ASE (Rescoring). The optimal geometric conformation of the best result was selected from the Ligand Interactions feature.

### 2.7. Expression and Purification of ZIKV RdRp

To harvest the protein expression of ZIKV RdRp, the ZIKV RdRp plasmids were transformed into Rosetta™ competent cells (Tiangen Biotech Co., Ltd., Beijing, China). Protein expression was induced with IPTG, and cells were harvested for purification with a Ni–NTA affinity column (His-trap HP, GE Healthcare, Beijing, China) according to the manufacturer’s suggested protocol. The concentration of a targeted protein was measured by SDS-PAGE using BSA (Sigma, St. Louis, USA) as a standard.

### 2.8. BIAcore Analysis

According to the protocol provided by the manufacturer, surface plasmon resonance (SPR) experiments were performed in a BIAcore T100 device (BIAcore Inc., Uppsala, Sweden) using CM5 sensor chips (GE Healthcare, Beijing, China). Briefly, recombinant ZIKV RdRp protein was immobilized on a CM5 chip. Different concentrations of doramectin or ribavirin were injected at a flow rate of 30 μL/min for 3 min. Subsequently, data were collected for a 3 min association followed by a 20 min dissociation. The chip was regenerated by injecting 10 μL of 15 mM NaOH for 20 s. All procedures were run in 1% DMSO PBS-P20 (GE Healthcare, Beijing, China) as a running buffer. The binding kinetics was analyzed with the software BIA-evaluation version 3.1 using a 1:1 Langmuir binding model. The K_d_ value was calculated as previously described [25].

### 2.9. Statistical Analysis

Statistical analysis was performed on triplicate experiments using a two-tailed Student’s *t*-test in the GraphPad Prism 8.0.2 software. The data results were expressed as the mean ± standard deviation (SD). *p* values were indicated by * *p* < 0.05, ** *p* < 0.01 or *** *p* < 0.001.

## 3. Results

### 3.1. Doramectin Exhibits Antiviral Potential without Cytotoxic Effects

**Figure 2:**
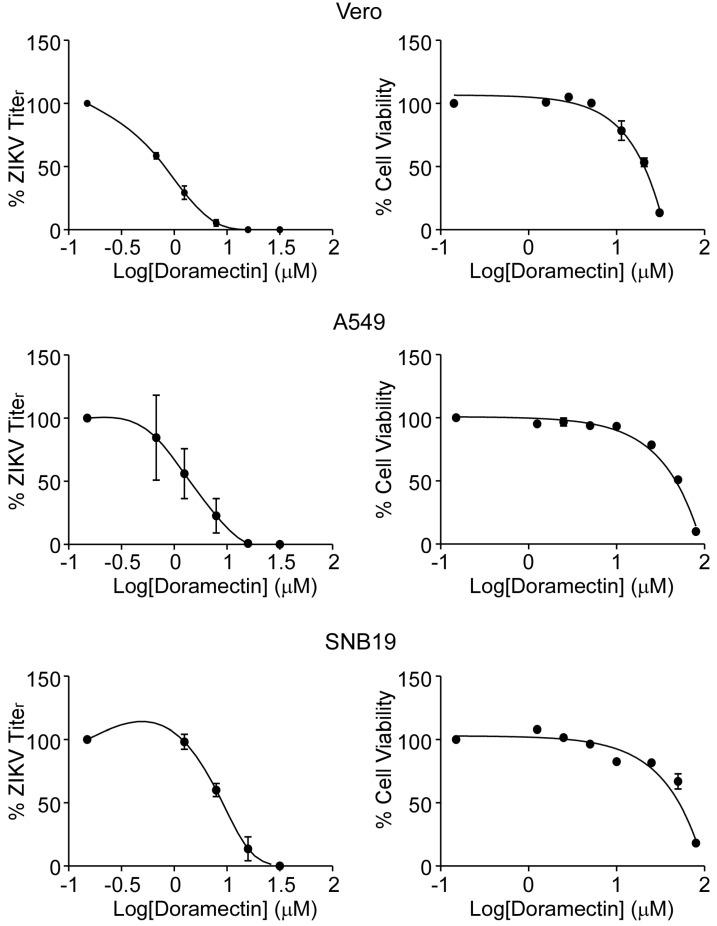
Antiviral activity and cytotoxicity spectrum of doramectin in Vero, A549, and SNB19 cells. ZIKV titer was quantified by plaque reduction assay and cell viability was detected by MTT assay.

To evaluate the antiviral effect of doramectin, a plaque forming assay was used to determine the EC_50_ with different concentrations (0.625, 1.25, 2.5, 5, 10 μM) of doramectin in Vero cells after ZIKV infection. The results showed that doramectin was effective against ZIKV, with an EC_50_ value of 0.85 ± 0.09 μM (Table 1), and showed relatively low cytotoxicity (CC50 = 50.7 ± 0.6 μM and SI =59.65). Moreover, we performed the same assay in A549 and SNB19 cells to verify that the antiviral effects were not specific to a single cell line. As we expected, doramectin also observably inhibited ZIKV infection both in A549 and SNB19 cells with EC_50_ values from 1.50~3.00 μM (Table 1). In addition, doramectin also exhibited significantly lower EC_90_ values (shown in Table 1) compared to its CC_50_ values (>50 μM) in the above three cell lines (Figure 2), indicating that doramectin could possess strong anti-ZIKV activity without causing significant cell cytotoxicity.

### 3.2. Doramectin Reduces the Expression of ZIKV Proteins

**Figure 3:**
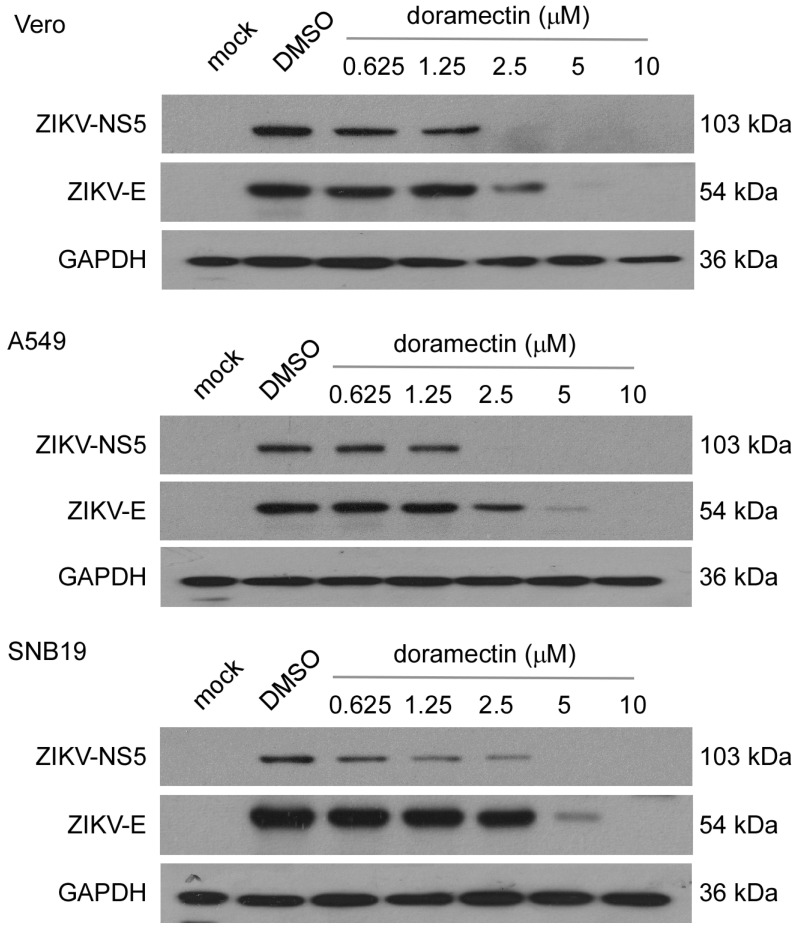
Doramectin inhibits the expression of ZIKV proteins. Western blotting analysis of protein expression of ZIKV NS5 and E in the cell lysates of Vero, A549, and SNB19 for the anti-ZIKV activity of doramectin or DMSO at indicated concentrations at 48 h post-infection (hpi) (MOI = 0.2).

To further explore the antiviral effect of doramectin on ZIKV, we detected the expression of two important ZIKV proteins in different cell lines by western blot. NS5 is the largest nonstructural protein of ZIKV and is critical for the replication of the ZIKV RNA genome. The E protein is involved in the fusion of the virus and host cell membranes. The results showed that the expression of both NS5 and E proteins decreased significantly after doramectin treatment in a dose-dependent manner (Figure 3). When the concentration of doramectin was 5 μM, ZIKV proteins barely expressed in Vero and A549 cells. However, only when the concentration of doramectin was 10 μM was the expression of these proteins completely inhibited in SNB19 cells (Figure 3). These results were consistent with the results of the EC_50_ and EC_90_ values against ZIKV in the above three cell lines.

### 3.3. Doramectin Prevents Cells from ZIKV Infection

**Figure 4:**
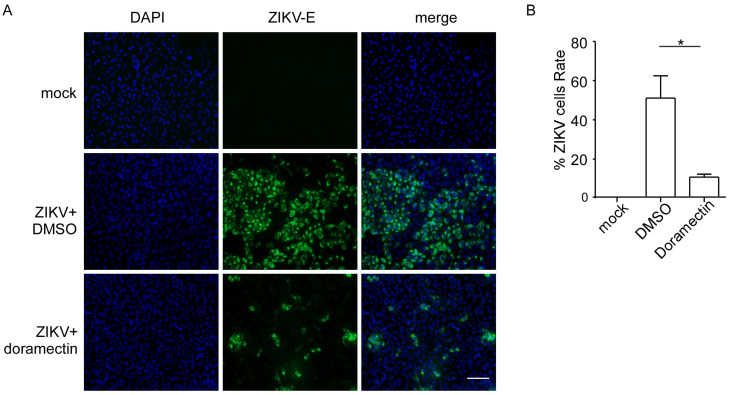
Doramectin prevents cells from ZIKV infection. (**A**) Immunofluorescence staining for ZIKV-E in ZIKV-infected A549 cells (MOI = 2) under the treatment by DMSO or 2.5 μM doramectin. Cell nuclei were stained with DAPI (blue). ZIKV-E protein was indicated in green (Scale bar, 100 μm). (**B**) Quantification of the number of ZIKV^+^ cells relative to mock infection is shown in the histogram. * *p* < 0.05.

To further determine the effect of doramectin against ZIKV infection, we used an immunofluorescence assay to visualize the antiviral effect of doramectin. As shown in Figure 4A, a large number of cells emitted green fluorescence in the control cells (ZIKV-infected with DMSO treatment), indicating a severe ZIKV infection. Additionally, the number of cells with green fluorescence decreased significantly after doramectin treatment, which signified a decrease in ZIKV-infection positive cells. The quantitative results also showed a statistically significant difference between the ZIKV group and doramectin group, suggesting that doramectin indeed effectively prevented ZIKV infection (Figure 4B).

### 3.4. Doramectin Binds Directly to the ZIKV RdRp

**Figure 5:**
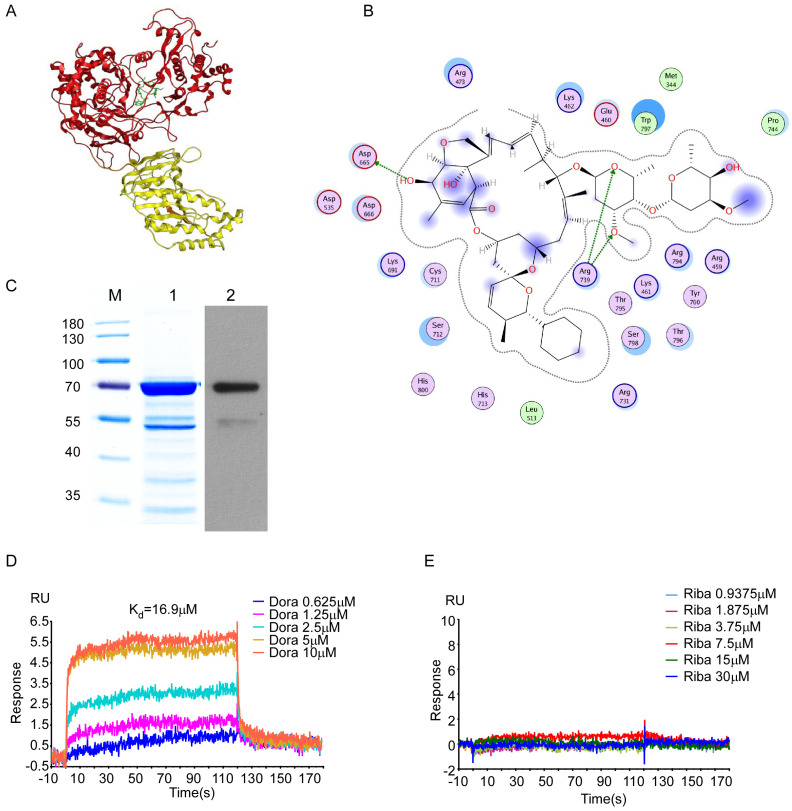
Doramectin directly binds to ZIKV RdRp. (**A**) Structural model for doramectin binding to the ZIKV NS5 protein was performed with Molecular Operating Environment (MOE). The NS5 protein is displayed as ribbons with the RdRp domain in red, MTase domain in yellow, and doramectin shown as green sticks. (**B**) Two-dimensional ligand-interaction maps of co-crystals of ZIKV NS5 domains bound with doramectin were generated using MOE. Polar residues are colored light purple, charged residues have an additional blue ring, and lipophilic residues are green. The degree of solvent exposure is shown by the blue halos. H-bond interactions to the amino acid side chain or main chain are shown as dashed green arrows, pointing toward the H-bond acceptor. (**C**) Coomassie blue-stained SDS-PAGE (lane 1) of crude extracts and western blot probed with anti-ZIKV NS5 (lane 2) after purification. (**D**,**E**) The surface plasmon resonance biosensor (SPR) assay to examine and characterize the binding of doramectin (**D**) or ribavirinc (**E**) to ZIKV RdRp. The K_d_ values were calculated via the BIAcore T100 analysis software (BIAevaluation Version 3.1).

To explore the specific antiviral mechanism of doramectin, we performed a molecular docking simulation analysis to investigate the potential binding between doramectin and ZIKV proteins. Three crystal structures of ZIKV proteins, including ZIKV NS1 (PDB: 5K6K), ZIKV NS2B-NS3 (PDB: 7VXX), and ZIKV NS5 (PDB: 5TFR), were downloaded from the Protein Data Bank (PDB) website. Furthermore, the Site Finder of Molecular Operating Environment (MOE) was used to identify the active structure of the protein domain as the docking site. As shown in Table 2, the docking scores were used to predict the binding affinity between compounds and protein targets, where higher binding affinity was defined by a more negative docking score. Notably, among the three potential targets, the docking score of doramectin and NS5 was the lowest (docking score = −40.33 kcal/mol), indicating that doramectin was most likely to interact with ZIKV-NS5. Additionally, in comparison with ribavirin (docking score = −13.92 kcal/mol), doramectin has a better affinity with NS5. Further analysis showed that doramectin binds to the “N” pocket with three H-bonds (Figure 5A,B). The “N” pocket is a pocket near the active site of the ZIKV RdRp domain that is essential for ZIKV genome RNA synthesis [26]. These results suggested that doramectin may dock with critical sites of ZIKV RdRp pockets and influence ZIKV genome RNA replication.

Next, we prepared the RdRp protein by purifying it from *Escherichia coli* (Figure 5C). Subsequently, the RdRp protein was immobilized on a CM5 sensor chip for the BIAcore assay based on the surface plasmon resonance biosensor (SPR) to investigate the direct interaction between doramectin and RdRp. The results showed that doramectin directly bound ZIKV RdRp protein (K_d_ = 16.9 μM), in contrast to the weak interaction between ribavirin and RdRp protein (Figure 5D,E), further suggesting the interaction indeed exists between doramectin and ZIKV RdRp.

## 4. Discussion

Thus far, there is still no specific antiviral agent or effective vaccine against ZIKV infection. The repurposing of existing drugs allows for the screening of simplified, convenient, and safe drug candidates and will facilitate pharmaceutical development. Here is the first study to demonstrate that the FDA-approved veterinary antiparasitic drug, doramectin, effectively inhibits ZIKV infection. The EC_50_ values of doramectin against ZIKV in different cell models were less than 3 μM, and the protein expressions of ZIKV NS5 and ZIKV E were repressed in ZIKV-infected cells. We further speculated that doramectin could inhibit the replication of ZIKV by binding to and blocking the key enzyme, RdRp, against ZIKV genome replication.

ZIKV infection can cause adverse clinical symptoms, such as microcephaly and miscarriage, Guillain-Barré syndrome, and so on. However, neither effective vaccines nor drugs have been approved for the prevention or treatment of ZIKV. Hence, it is still worth our attention to develop potent, efficacious, and safe anti-ZIKV drugs. To date, a variety of ZIKV inhibitors have been reported, such as JMX0207 [11], tetrapeptide-boronic acid [27], hydroxychloroquine [28], myricetin [29], methylene blue [30], and erythrosin B [31], which can inhibit the activity of the NS2B-NS3 enzyme to inhibit ZIKV infection. Furthermore, there are peptide inhibitors targeting the ZIKV E protein to inhibit virus invasion [32], and chloroquine compounds targeting the ZIKV membrane fusion process [33]. Moreover, ZIKV-RdRp plays an important role in the virus life cycle by regulating virus replication and is a significant target for anti-ZIKV screening [34]. Previous studies have reported that NS5-RdRp inhibitors such as favipiravir [35], NITD-008 [36], ribavirin [37], and sofosbuvir [38] directly penetrate into the replication chain due to their structural similarities to nucleotides, terminating the synthesis of ZIKV RNA. Unlike these compounds, doramectin, a macrocyclic lactone, was shown to directly bind to ZIKV-RdRp by SPR. In addition, further in silico analysis indicated an interaction between doramectin and the “N” pocket of RdRp, which is similar to the combination of fidaxomicin and ZIKV RdRp in our previous study [39]. Accordingly, we postulate that the binding between doramectin and ZIKV RdRp may be related to the effect on ZIKV replication. Because the RdRp sequence of flaviviruses is conserved, whether doramectin may also inhibit the replication of flaviviruses such as DENV and YEV by a similar mechanism requires further investigation.

No compounds against ZIKV have entered clinical trials at present. The FDA-approved drugs, such as ribavirin and sofosbuvir, are contraindicated for pregnant women since they may cause congenital malformation or developmental delay [40,41]. However, the patients with severe adverse outcomes after ZIKV infection are mainly pregnant women. These drug contraindications limit clinical trials and applications. Therefore, it is still a major challenge to discover potent anti-ZIKV drugs suitable for pregnant women and infants. Drug repurposing screens have recently emerged as an alternative approach to accelerate drug development [42,43]. Following a repurposing phenotypic screen, new indications for existing drugs may be rapidly identified, and clinical trials can be conducted quickly. Hence, repurposing approved drugs is one of the effective strategies to develop anti-ZIKV drugs. Previously, doramectin was mostly used for animal disease therapy. There have been several published studies demonstrating the successful treatment and prevention of pregnant animals from parasite burdens in calves [44,45]. These results provided a possibly tolerable safety profile for future applications of this agent as a clinical anti-ZIKV drug, which may be suitable for pregnant women and infants. In this context, further effort in implementing structural optimization and transformation is needed in future investigations.

In conclusion, our findings demonstrate that a widely used veterinary medication approved by the US FDA, doramectin, is a potent agent against ZIKV infection through directly targeting the RdRp of ZIKV. This study provides a new treatment option for ZIKV-associated diseases and a promising drug candidate for treating patients with ZIKV infection.

## Figures and Tables

**Table 1:** Efficacy of doramectin against ZIKV in 3 different cell lines.

Cell Line	EC_50_ (μM) ^1^	EC_90_ (μM) ^2^	CC_50_ (μM) ^3^	SI ^4^
Vero	0.85 ± 0.09	2.28 ± 0.09	50.7 ± 0.6	59.65
A549	1.50 ± 1.15	3.38 ± 0.42	60.1 ± 3.4	40.07
SNB19	3.00 ± 0.17	6.29 ± 2.17	50.4 ± 3.4	16.80

^1^ The 50% effective concentration, or the concentration necessary to reduce viral yield by 50%, was determined using a plaque assay. ^2^ The 90% effective concentration, or the concentration necessary to reduce viral yield by 90%, was determined using a plaque assay. ^3^ Concentration required to inhibit the cell growth by 50% in the absence of virus. ^4^ Selectivity index, SI = CC_50_/EC_50_.

**Table 2:** The Docking scores (Kcal/mol) of doramectin against ZIKV NS1, NS2B-NS3, and NS5. Values were obtained using MOE software.

Compound	Docking Score (kcal/mol)		
	ZIKV-NS1(5K6K)	NS2B-NS3 (7VXX)	NS5 (5TFR)
doramectin	−10.90	−10.26	−40.33
ribavirin	−6.80	−7.40	−13.92

## Data Availability

The data presented in this study are available on request from the corresponding author.
